# Aquablation versus HoLEP in patients with benign prostatic hyperplasia: a comparative prospective non-randomized study

**DOI:** 10.1007/s00345-024-04997-0

**Published:** 2024-05-09

**Authors:** Jakob Michaelis, Max Träger, Sophie Astheimer, Moritz von Büren, Elfi Gabele, Markus Grabbert, Jan Halbich, Marius Kamps, Jonas Klockenbusch, Theresa Noll, Phillippe Pohlmann, Daniel Schlager, August Sigle, Martin Schönthaler, Konrad Wilhelm, Christian Gratzke, Arkadiusz Miernik, Dominik Stefan Schöb

**Affiliations:** Department of Urology, Faculty of Medicine, Medical Centre – University of Freiburg, Hugstetter Str. 55, 79106 Freiburg, Germany

**Keywords:** Aquablation, Endourology, HoLEP, BPS

## Abstract

**Purpose:**

The question of best surgical treatment for lower urinary tract symptoms (LUTS) due to benign prostate hyperplasia (BPH) remains controversial. We compared the outcomes of aquablation and holmium laser enucleation of the prostate ("HoLEP") in a prospective cohort.

**Methods:**

Patients with BPH underwent aquablation or HoLEP according to their preference between June 2020 and April 2022. Prostate volume (“PV”), laboratory results, postvoid residual volume, uroflowmetry, IPSS, ICIQ-SF, MSHQ-EjD, EES and IIEF were evaluated preoperatively and at three, six and 12 months postoperatively. We also analyzed perioperative characteristics and complications via the Clavien Dindo („CD“) classification.

**Results:**

We included 40 patients, 16 of whom underwent aquablation and 24 HoLEP. Mean age was 67 years (SD 7.4). Baseline characteristics were balanced across groups, except the HoLEP patients’ larger PV. IPSS fell from 20.3 (SD 7.1) at baseline to 6.3 (SD 4.2) at 12 months (p < 0.001) without differences between aquablation and HoLEP. HoLEP was associated with shorter operation time (59.5 (SD 18.6) vs. 87.2 (SD 14.8) minutes, p < 0.001) and led to better PV reduction over all timepoints. At three months, aquablation’s results were better regarding ejaculatory (p = 0.02, MSHQ-EjD) and continence function (p < 0.001, ICIQ-SF). Beyond three months, erectile, ejaculatory, continence function and LUTS reduction did not differ significantly between aquablation and HoLEP. CD ≥ grade 3b complications were noted in six patients in aquablation group while only one in HoLEP group (p =  < 0.01).

**Conclusions:**

While aquablation revealed temporary benefits regarding ejaculation and continence at three months, HoLEP was superior concerning operation time, the safety profile and volumetric results.

**Supplementary Information:**

The online version contains supplementary material available at 10.1007/s00345-024-04997-0.

## Introduction

Benign prostatic hyperplasia (BPH) is a highly prevalent condition in older men that triggers lower urinary tract symptoms (LUTS) [1]. Although medical treatment can alleviate symptoms [2], a significant proportion of patients needs surgical therapy to enable a lasting resolution of their symptoms [3]. There is a plethora of operation methods for BPH.

Aquablation is one of the novel options that has rapidly gained in importance. It functions via hydrodissection, by which a high-velocity water jet ablates prostate tissue, thus representing a mechanoablative method in contrast to its widely used thermoablative alternatives.

Aquablation has advantages in terms of morbidity, especially regarding preserving erectile function and prograde ejaculation when compared to TUR-P [4, 5]. Another key advantage is the short operative time, outperforming different laser enucleation techniques as well as photovaporization of the prostate [6]. Recent trials on aquablation reported promising long-term data with low rates (6.0% and 3.7%) of additional BPH therapy required until the fifth year post-therapy [7, 8]. Aquablation’s learning curve is also short, achieving a predefined trifecta outcome (operative time < 60 min plus hemoglobin loss limited to under 2g/dl plus avoiding CD > grade 2 complications) in 70% of the first 50 patients [9]. On the downside, the inability to histologically evaluate the ablated tissue poses a crucial limitation of the aquablation procedure [10].

Aquablation has so far only been compared in detail to transurethral resection of the prostate (TUR-P) [11]. TUR-P is associated with a significant range of complications [12, 13], i.e., urethral or bladder neck strictures. However, aquablation is also known to cause relevant complications, for example postoperative bleeding complications or—rarely—rectal perforation [14]. Postoperative bleeding was particularly evident in conjunction with very large prostates [15] or when cauterization was deliberately omitted [16]. Bleeding complications were significantly reduced in subsequent trials by focal cauterizing of the bladder neck at the end of the procedure [17, 18]. Multi-pass aquablation appears to be more effective than single-pass aquablation with regard to volume reduction [19].

Comparisons between aquablation and techniques other than TUR-P are scarce. Holmium laser enucleation of the prostate (HoLEP) is one of the most frequent surgical treatments [20] for BPH. HoLEP yields favorable outcomes in comparison to TUR-P in uroflowmetric results, retreatment rates, and in patient-reported outcomes [21]. Comparative analyses between HoLEP and aquablation are lacking except for assessments of perioperative bleeding complications [22]. We devised/planned this project to address the knowledge gap concerning objective and patient-reported outcomes, perioperative characteristics, and safety when comparing HoLEP and aquablation.

## Materials and methods

Patients with BPH were either scheduled for aquablation or HoLEP according to patient preference following urological consultation providing information on therapy options. During their preoperative work-up, all patients were offered extended follow-up as part of this prospective study. Exclusion criteria were a history of prostate cancer, indwelling urinary catheterization for longer than 3 months (to ensure reliable information on preoperative continence and prostate-related symptoms), urethral strictures, bladder stones, chronic pelvic pain syndrome, antimuscarinergic therapy due to overactive bladder, and anticoagulation unable to be discontinued.

Aquablation was performed in a standardized fashion, resembling the approach described by Zorn et al. [23]. After resection, patients regularly underwent hemostasis via monopolar diathermy for focal bladder neck cauterization. HoLEP was conducted using the three horse shoe-like incision technique [24]. A group of three surgeons (A.M., M.S. and K.W.) with at least 5 years of HoLEP experience and each having conducted over 100 procedures performed HoLEP. Aquablation was executed by a fixed team of two surgeons (A.M. and D.S.S.) with one year of experience each and 9 cases performed together prior to the first patient included in this analysis.

Baseline evaluation and follow-up at three, six and 12 months after surgery incorporated the five questionnaires IPSS, International Index of Erectile Function (IIEF), International Consultation on Incontinence Questionnaire – short form (ICIQ-SF), Male Sexual Health Questionnaire—ejaculatory dysfunction (MSHQ-EJD) and Ejaculation, Erection and Satisfaction Scale (EES). Besides those, we carried out uroflowmetry and transrectal ultrasound (TRUS) volumetry (calculated by [height x length x width x π/6]) and monitored PSA values and creatinine. Operation time was measured from the surgeon’s first preparation step at the operating table until the final catheter insertion for both interventions. We recorded the maximum perioperative daily pain level via a numerical rating scale (verbal NRS-11). Time to catheter removal, sonographic post-void residual volume and time to discharge were also documented.

We conducted statistical analyses using Pearson's chi-squared test for categorical, two sample t-test for normally distributed continuous and Wilcoxon signed-rank test for non-normally distributed continuous variables. We decided against applying methods for imputation of missing values. Analyses were done with R language and environment for statistical computing (version 4.3.0, R Foundation for Statistical Computing, Vienna, Austria). Significance level was set at 0.05.

## Results

In all, 58 patients declared their initial interest in participating, of whom 18/58 were excluded (14 aquablation, four HoLEP). 8/58 (6/8 aquablation, 2/8 HoLEP) were excluded preoperatively because of failing to complete the baseline questionnaires. Ten of the remaining 50 (8/10 aquablation, 2/10 HoLEP) patients withdrew their consent either preoperatively or by the first follow-up appointment. This was mainly due to Covid-19 pandemic restrictions or because patients were no longer willing to comply with extended follow-up. Our final evaluation included 40 patients.

We included these patients between June 2020 and April 2022; 16 subjects underwent aquablation and 24 the HoLEP procedure. Mean age was 67 years (range 52 – 82; SD 7.4), patients presented with a prostate volume (PV) of 66.3 cm^3^ (range 33 to 118 cm^3^, SD 20.9) and mean PSA value of 4.87 (SD 4.18) ng/ml. Mean IPSS of 20.3 (median 22) at baseline reflected the severity of BPH-related symptoms. Baseline characteristics were adequately balanced across groups, except that HoLEP patients presented a larger PV (73.8 cm^3^ ± 18.1 SD vs. 55.0 cm^3^ ± 19.2 for aquablation, p = 0.005). Participation in the three, six, and 12-month follow-ups ranged from 85 to 95 percent (further baseline characteristics are summarized in Table 1).

**Table 1:** Patient baseline parameters. Note: Anticoagulation had to be paused preoperatively

	All	Aquablation	HoLEP	*p*-value
Patient number	40	16	24	
Age (years)	67.1	66.9	67.3	0.87
BMI (kg/m^2^)	26.5	26.7	26.4	0.70
Prostate volume [ccm]	66.3	55.0	73.8	**0.005**
PSA [ng/ml]	4.87	3.75	5.62	0.17
Anticoagulation	0.15	0.06	0.21	0.22
Post-void residual volume [ml]	106.0	110.2	102.9	0.84
Maximum flow rate [ml/s]	11.9	11.3	12.2	0.56
IPSS	20.3	21.3	19.8	0.53
llEF	20.4	21.4	19.7	0.52
ICIQ-SF	5.1	6.4	4.3	0.19
MSHQ-EjD	9.6	9.9	9.4	0.77
Patient number 3 month postoperative	38	14	24	
Patient number 6 month postoperative	35	16	19	
Patient number 12 month postoperative	34	13	21	

Significant values are printed in bold

**Fig. 1:**
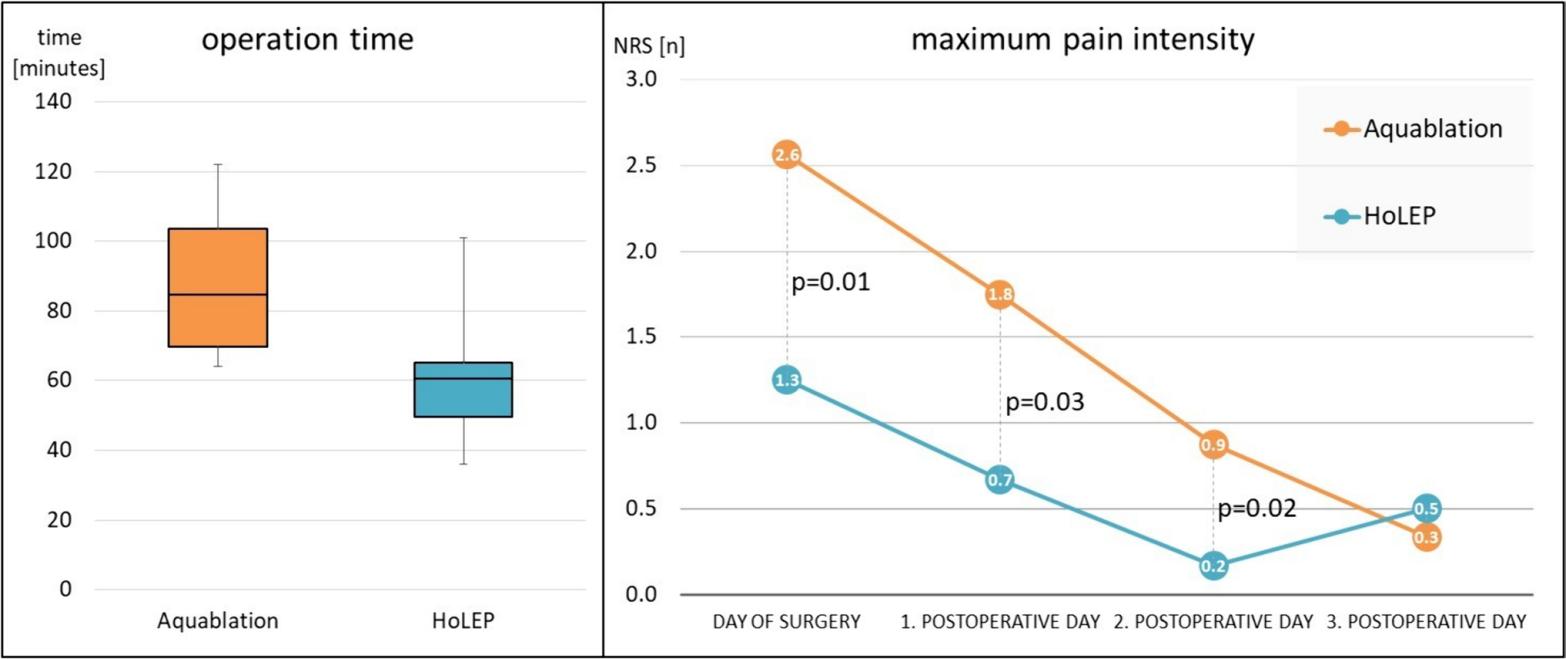
Perioperative characteristics

Average operation time for aquablation was significantly longer than for HoLEP (87.2 ± 14.8 vs. 59.5 ± 18.6 min, respectively; p < 0.001). Aquablation’s maximum pain intensity was also higher than HoLEP’s, with the pain maximum on numeric rating scale of 2.6 ± 1.6 vs. 1.3 ± 1.5 (p = 0.01), 1.8 ± 1.8 vs. 0.7 ± 1.2 (p = 0.03) and 0.9 ± 1.3 vs. 0.2 ± 0.6 (p = 0.02) on the day of surgery, and first and second postoperative day, respectively. Nevertheless, pain intensity was generally low with no patient requiring escalating analgesics to opioids (see Fig. 1). We found no significant differences in time to catheter removal (median two days) or time to discharge (median three days) between the groups.

**Fig. 2:**
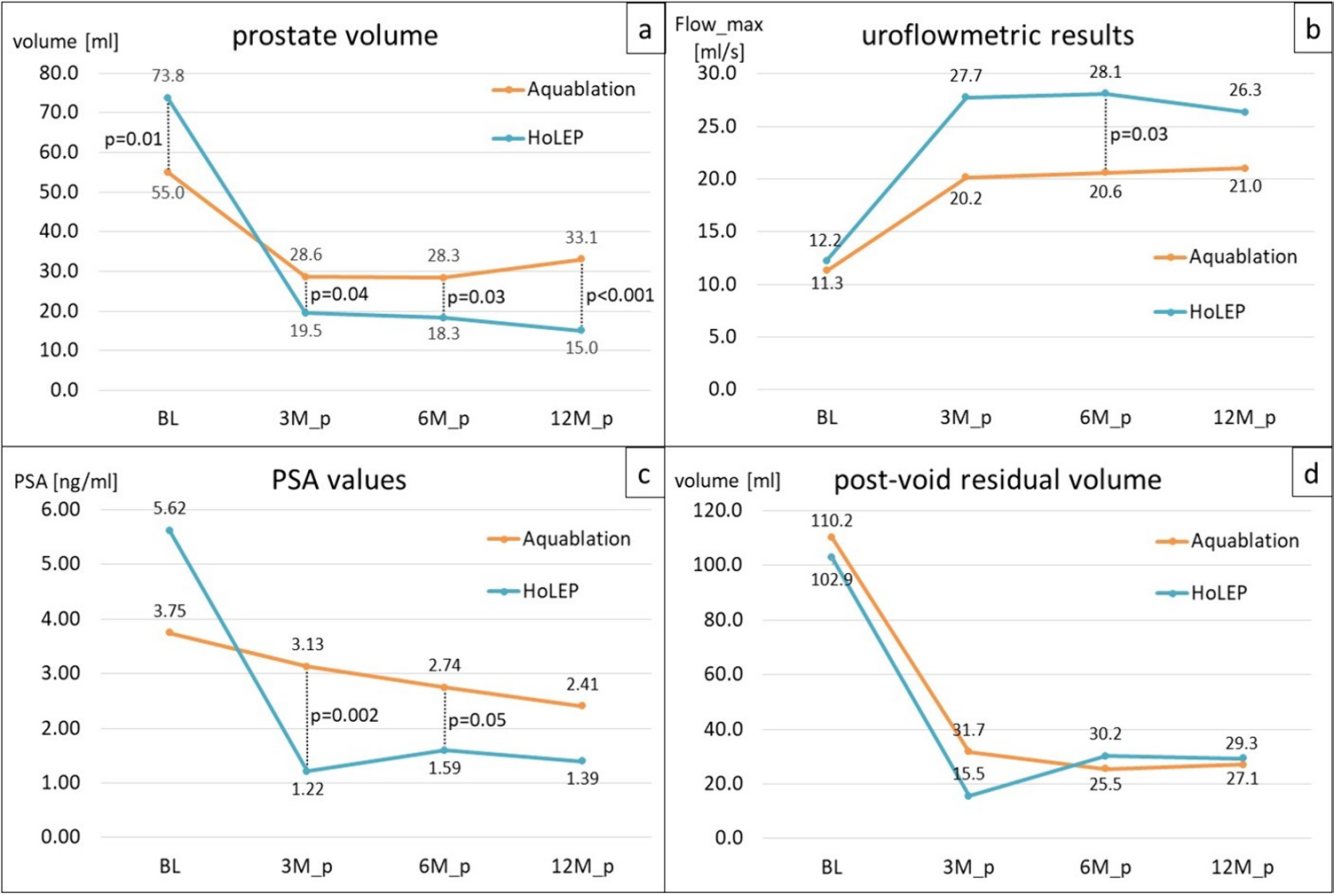
Objective outcomes at baseline (BL) and at follow-up examinations at three months postoperative (3M_p), six months postoperative (6M_p) and 12 months postoperative (12M_p), respectively. PV was measured via transrectal sonography (**a**), uroflowmetric outcomes are reported as maximum urinary flow rate (**b**) with sonographic evaluation of post-void residual volume afterwards (**d**). PSA values are measured as serum levels by chemiluminescence immunoassay (**c**)

At all follow-up timepoints, HoLEP’s relative PSA-reduction was significantly greater than aquablation’s ( – 78%/ – 72%/ – 75% vs.  – 16%/ – 27%/ – 36% at 3, 6 and 12 months, respectively). We observed no significant changes in creatinine levels over time. Differences in post-void residual volume never reached statistical significance at any follow-up timepoint with 21.0 ± 43.6 ml for aquablation and 22.4 ± 54.1 ml for HoLEP mean post-void residual volume across all follow-up examinations (p = 0.86) compiled. PV reduction in our HoLEP patients, was markedly larger at all follow-up timepoints when compared to aquablation patients. Residual volume at 12 months was 15.0 ± 5.1 cm^3^ ( – 79.6% compared to preoperative volumetry) for HoLEP, while aquablation-treated patients had glands of 33.1 ± 11.9 cm^3^ ( – 39.2%) (p < 0.001). Uroflowmetric outcomes favored HoLEP, reaching statistical significance at six months postoperative (maximum urinary flow rate 28.1 ± 11.3 ml/s for HoLEP vs. 20.6 ± 6.7 ml/s for aquablation, p = 0.03). Detailed results are illustrated in Fig. 2.

IPSS improved significantly over time, dropping from 21.3 ± 7.9 (median 23) vs. 19.8 ± 6.5 (median 21) at baseline to 9.1 ± 4.8 (median 10) vs. 10.5 ± 4.5 (median 12), 8.1 ± 5.8 (median 6) vs. 7.9 ± 4.5 (median 6) and 6.5 ± 4.4 (median 5) vs. 6.2 ± 4.1 (median 5.5) at three, six and 12 months for aquablation vs. HoLEP without differences across treatment groups. Concerning quality of life regarding prostate symptoms obtained in IPSS, both groups improved from 4.1 ± 1.5 vs. 3.8 ± 1.3 (median 4 each) at baseline to 1.6 ± 1.5 vs. 1.2 ± 0.7 (median 1 each) for aquablation vs. HoLEP, also without significant differences between groups. Postoperative ejaculative function showed significant deterioration for HoLEP, but not aquablation. That difference favoring aquablation was significant at three months after surgery and remained a non-significant trend at later timepoints. At three months, continence was significantly worse for HoLEP than aquablation, but continence status recovered by the six-month post-operative follow-up. Both groups reported similar erectile function outcomes with no statistically significant difference. For more details about functional outcomes, see supplementary Fig. 3.

We observed a significant higher number of severe complications in association with aquablation compared to HoLEP, with Clavien-Dindo ≥ grade 3 complications in six (37.5%) vs. one (4.2%) patient (p < 0.01). This was mainly driven by their higher rate of postoperative bleeding requiring revision. (One case of iatrogenic rectal perforation occurred, which was managed by intraluminal vacuum therapy and protective ileostomy on postoperative days 5 and 6, respectively. That patient underwent an ileostomy reversal and ileoileostomy four months postoperatively. Surgical retreatment due to persistent/recurrent bladder outlet obstruction was necessary in one patient in each group. No blood transfusions were necessary in either group. Minor complications (Clavien-Dindo grade 1–2) were more frequent for HoLEP without reaching statistical significance (8/24 for HoLEP vs. 2/16 for aquablation, p = 0.14), particularly attributable to their higher rates of urinary retention. Supplementary Fig. 4 shows details about each group’s complications and their severity over time group.

## Discussion

In this clinical study, we compared the efficacy and safety of aquablation and HoLEP as treatment modalities for benign prostatic hyperplasia (BPH). The study included a one-year follow-up period to assess the long-term outcomes of these procedures.

We found no significant differences in IPSS, uroflowmetric results and postvoid residual volume at the final one-year follow-up. However, PV reduction was significantly better in the HoLEP group, which, at least at 6 months, also translated into significantly better uroflowmetric results. Concerning PV reduction, HoLEP as a procedure guided by the anatomical limits of the surgical capsule appears to have advantages over a TRUS controlled procedure like aquablation, even when the latter is combined with “fluffy tissue” resection and focal bladder neck cauterization. As decision about single vs. multi-pass aquablation was not specified by our study protocol and evidence for the beneficial effects of multi-pass aquablation arised recently [19], it will be interesting to see if the PV reduction continue to differ significantly with mandatory multi-pass aquablation in other cohorts. Our cohort’s PV reduction, IPSS improvement and improvement in maximum urinary flow rate of aquablation were similar to other studies: Whiting et al. reported a residual PV of 33.2 cm^3^, IPSS of 6.1 and maximum urinary flow rate of 23.9 ml/s at 12 months [25], which is well in line with our results (33.1 cm^3^, 6.5 points and 21.0 ml/s, respectively).

As reported previously, the aquablation group showed an advantage regarding preservation of ejaculative function (difference in MSHQ-EjD 4.2 points at three months favoring aquablation (p = 0.021) vs. HoLEP in our study compared to 3.1 points difference favoring aquablation vs. TUR-P at 3 months (p = 0.002) in Gilling et al. 2019 [26]). Nevertheless, these differences in MSHQ-EjD did not maintain significance beyond the three-month time point. Postoperative erectile function was also similar in both groups. Regarding patient safety: our HoLEP results were favorable, with a significantly higher number of severe complications in the aquablation group, especially their higher rate of postoperative bleeding. This finding is supported by other studies, showing a significant higher loss of hemoglobin and transfusion rates for aquablation compared to transurethral resection [27] and as a non-significant trend for transurethral enucleation [22]. Despite that, none of our patients required a blood transfusion (compare to the 5,9% transfusion rate in the WATER-II trial) [28]. We guess that our surgical therapy of postoperative bleeding to avoid blood transfusion was more aggressive, which may explain these different results. However, we observed no differences in the occurrence of complications up to 12 months.

Aquablation’s significantly longer operation time in our study differs from previous reports, as e.g., 45, 38, and 37 min, respectively [11, 22, 29]. We defined operation time as the surgeon’s start of intervention preparations at the operating table up to catheter insertion. We found various definitions of operation times in other studies, e.g. handpiece placement to final urinary catheter placement [29], pre- to post treatment cystoscopy [11] or TRUS insertion to urinary catheter placement [28]. As none of these definitions consider all the preparation steps, they may fail to accurately measure total procedure time. We believe our definition reflects more accurately the genuine amount of time these procedures take. Our definition should at least partially account for our prolonged mean operation times for aquablation in our study. Another reason for our longer durations per patient might be partly that we had performed very few (nine aquablations) in our institution before initiating this study, while we had extensive experience (> 750 cases) with HoLEP. This may have also affected other outcomes variables and should be taken into account, even if previous studies proved the short learning curve for aquablation [9] concerning safety and operative time. Shorter procedure times are obviously a medical advantage for patients, and there is also a potential benefit for health care providers (since one can do more procedures within the same time span), as well as for the healthcare system (as more patients being treated by one center may reduce time to surgery and thus improve regional medical care). We can conclude that both procedures have their specific strengths and that to offer a broad range of different procedures might give clinicians the opportunity to offer each patient the procedure which suits best their individual priorities and objectives.

There are several limiting factors to our study to be considered: Above all, the relatively small number of participants limits our results ‘ generalizability. The non-randomized study design can lead to selection bias. This may be reflected in the difference in baseline volume between the groups, even if TRUS volumetry has known inaccuracies. Different baseline volumes might also have affected other parameters such as operating time, bleeding rate, PSA values or uroflowmetric outcomes. Moreover, patients’ compliance with aftercare appointments was impaired due to the Covid-19 pandemic, which led to a 15% loss of follow-up data, which might detract from the robustness of our findings. Distinct levels of experience with the two procedures could also have influenced our results. Long-term follow-ups beyond one year are necessary to assess the durability of outcomes and potential late complications associated with both aquablation and HoLEP. Further studies with a higher case load are necessary to provide more data on the benefits and disadvantages of both procedures. Prospective trials comparing HoLEP and aquablation are already recruiting (NCT04801381, NCT04560907 [30]).

## Conclusions

Both HoLEP and aquablation are effective treatment options for patients with LUTS due to BPH. The choice between these techniques should be individualized, considering each patient's specific needs and preferences, as well as the surgeon's expertise. Our study data suggest no functional advantage for either of these procedures beyond three months. However, HoLEP’s safety profile and volumetric results proved to be superior. The generalizability of our study findings is restricted by several limitations. Long-term follow-up and patient-reported outcomes assessments will further enhance our understanding of the outcomes and patient satisfaction associated with these interventions to better advise our patients.

## Supplementary Information

Below is the link to the electronic supplementary material.

Supplementary file1 (JPG 180 KB)

Supplementary file2 (JPG 66 KB)
