# Supplementary figures and images for: Aquablation versus HoLEP in patients with benign prostatic hyperplasia: a comparative prospective non-randomized study
Source: World J Urol. 2024 May 9;42(1):306. doi: 10.1007/s00345-024-04997-0 (PMC11081982; doi:10.1007/s00345-024-04997-0)

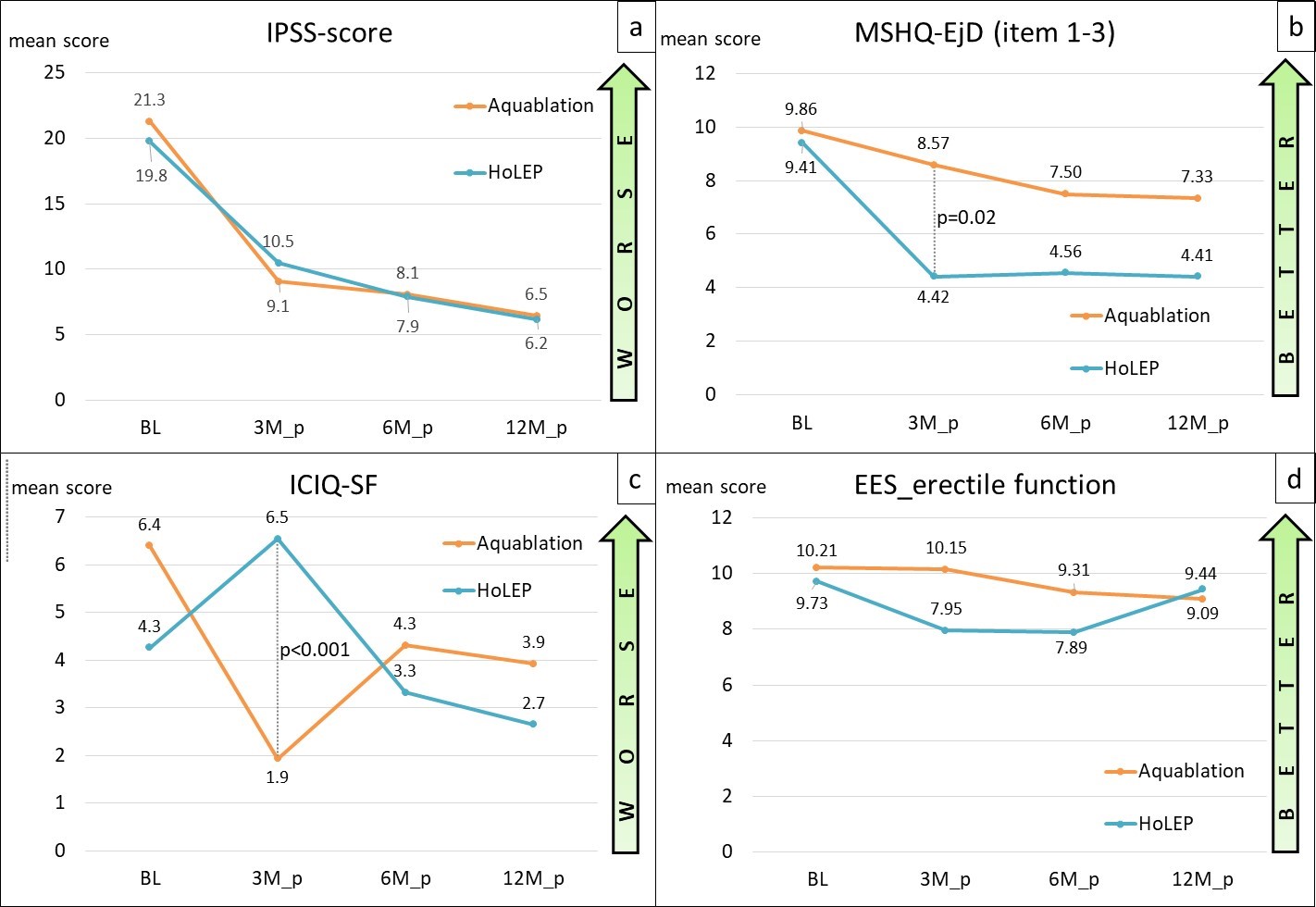

Supplement: Supplementary file 1 — Supplementary file1 (JPG 180 KB) [file 345_2024_4997_MOESM1_ESM.jpg]

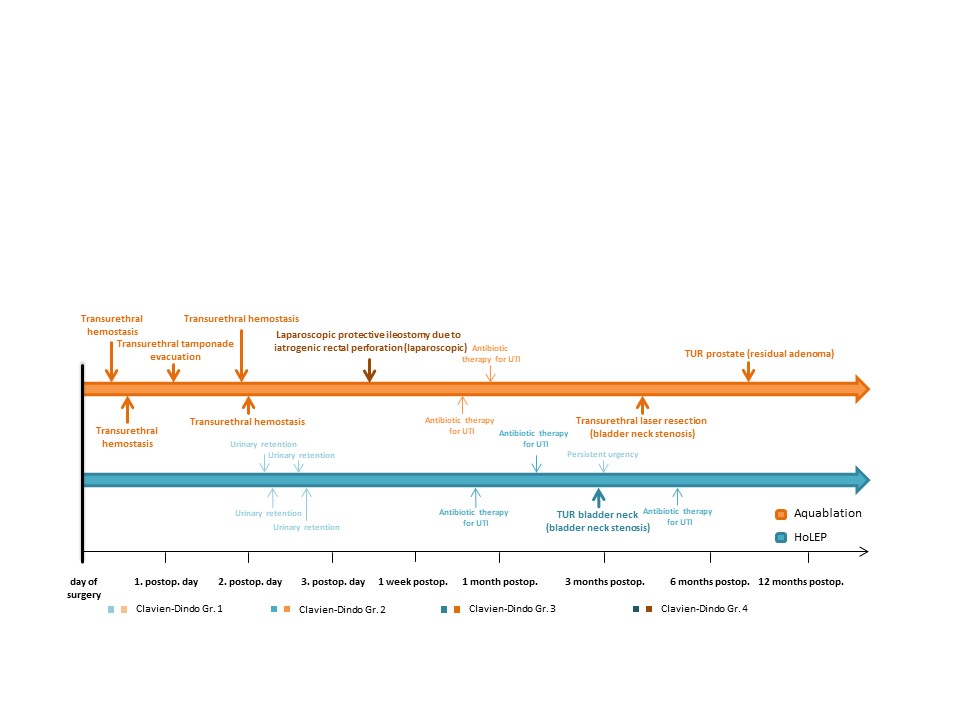

Supplement: Supplementary file 2 — Supplementary file2 (JPG 66 KB) [file 345_2024_4997_MOESM2_ESM.jpg]
